# A Urachal Cyst Case with Painful Mass Locates at Ileal Mesentery

**DOI:** 10.1155/2015/240362

**Published:** 2015-12-13

**Authors:** Selahattin Koray Okur, Hüseyin Pülat, Oktay Karaköse, Ismail Zihni, Kazım Çağlar Özçelik, Hasan Erol Eroğlu

**Affiliations:** ^1^Department of General Surgery, Suleyman Demirel University Faculty of Medicine, 32260 Isparta, Turkey; ^2^Department of Surgical Oncology, Suleyman Demirel University Faculty of Medicine, 32260 Isparta, Turkey

## Abstract

Urachal cyst is an unusual clinical condition, which is usually asymptomatic. In some adult cases, it may lead to complications. The cyst is between umbilicus and urinary bladder. It is diagnosed via ultrasonography and computed tomography. However, in some cases, the diagnosis is made by means of surgical exploration and histopathological evaluation. In this paper, we report a case of a 17-year-old female presenting with painful abdominal mass. At the first evaluation, the case was diagnosed as a mesenteric cyst because the mass located in the mesentery, and final histopathological report revealed the urachal cyst.

## 1. Introduction

The urachus is an embryonic connection between the urinary bladder and the allantois. It obliterates during early infancy, to form the median umbilical ligament between the transverse fascia and the peritoneum. Histologically, the inner layer is modified transitional epithelium, the middle is fibroconnective tissue, and the outer layer is a smooth muscle layer [1]. If urachus does not obliterate, urachal anomalies may appear, such as patent urachus, urachal sinus, urachal diverticula, urachal cyst, and urachal cord [2]. In this paper we report the case of 17-year-old female who has been diagnosed with urachal cyst.

## 2. Case Report

**Figure 1:**
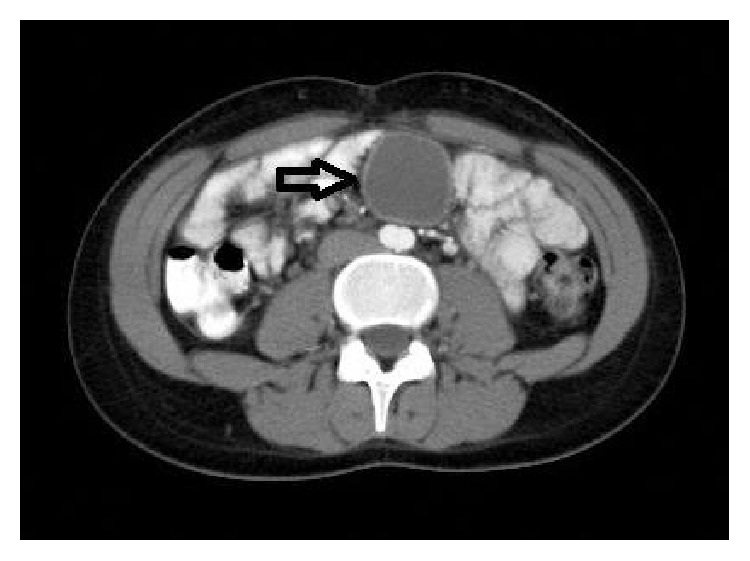
Tomographic view of the urachal cyst (arrow).

**Figure 2:**
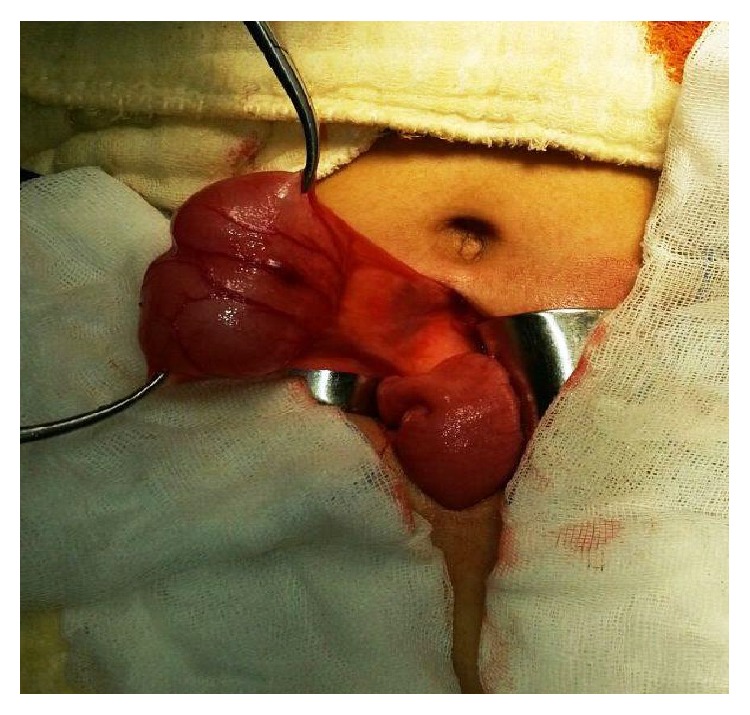
Intraoperative view.

**Figure 3:**
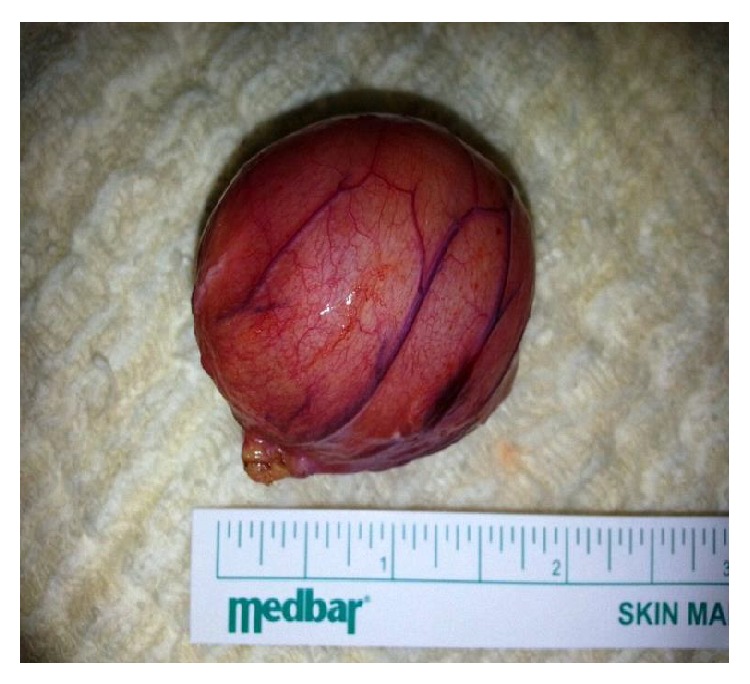
The appearance of the mass.

A 17-year-old female suffered from periumbilical pain. She had a history of tonsillectomy and was still taking pantoprazole for epigastric pain. There was no complaint related to the urinary system. In the physical examination, there was a palpable painful mass, approximately 5 cm in diameter, at the posterior of the umbilical region. The laboratory tests were within normal limits. On abdominal ultrasonography (US) and abdominal computed tomography (CT), a cystic mass was seen in the posterior of the umbilical region, extending towards the left side, 41 × 39 mm in size (Figure 1). With these findings, a diagnostic minilaparotomy was performed. During surgery, a 4 cm diameter cystic mass connected with the ileal mesentery was identified (Figures 2 and 3). Neither umbilicus nor urinary bladder had a connection with the cystic mass. After total excision of the mass, the pathological evaluation was reported as urachal cyst. The patient was discharged with cure on postoperative second day. At the third month follow-up examination, no complication was observed.

## 3. Discussion

The most frequent form of urachal anomalies is urachal cyst at rates of 30% and it is more frequent in males. Unless complications develop in urachal cysts, they are small, silent, and asymptomatic. Complications may include infection, bleeding within the cyst, enlargement, intraperitoneal rupture, intestinal fistula, intestinal obstruction, lithiasis, and a high incidence of malignant degeneration [3, 4]. There may also be concomitant urinary tract infection, macroscopic hematuria, or dysuria. Although US is known to be the most common and cost-effective diagnostic tool, radiological imaging methods such as CT and MR are also used [5].

In the differential diagnosis, vitelline duct anomalies, appendicitis, granulomatous inflammations, and granulation tissue from the umbilical stump should be kept in mind [6]. In our patient, the cyst locates at ileal mesentery. Mesenteric cysts are usually localized in ileal mesentery (60%). Its incidence has been reported approximately in 1/100000, twice in females. The mesenteric cysts are divided into embryonal, traumatic, neoplastic, and infectious types according to etiological and clinical features. Histopathological classification includes lymphatic, mesothelial, enteric, urogenital, and dermoid cyst and nonpancreatic pseudocyst. They are asymptomatic until old age without complications [7, 8].

In the treatment of urachal anomalies in adults, recurrence occurs at rates of 31% when treatment is made by drainage rather than by conservative treatment [9]. In addition, in cases of residual tissue, there is a risk of malignancy. In a study by Ashley et al. [10] in which 130 adult patients with urachal anomaly were evaluated, malignancy was found at the rate of 51% and age over 55 years and the presence of hematuria were found to be strong predictors. Therefore, total excision is recommended. In the case presented here, total excision was applied with minilaparotomy. Mesenteric cyst and urachal cysts were compared in Table 1.

## 4. Conclusion

Although urachal anomalies are rarely seen, complete surgical application is required due to the high incidence of recurrence and malignancy in adult patients. Despite pediatric surgery and urology clinic interventions, when there is a urachal cyst, as in the current case, it is one of the probable pathologies encountered by general surgeons as an intra-abdominal mass. Sometimes, its diagnosis is possible with surgical exploration or histopathological evaluation like our patient.

## Figures and Tables

**Table 1:** Comparison of mesenteric cyst and urachal cysts.

	Urachal cyst	Mesenteric cyst

Incidence	Unclear	1/100000
Sex	Men > women	Women > men
Age	20–40	Second decade
Asymptomaticity	Usually	Usually
Most complications	Infection	Inflammation
CT findings	Cystic mass between transverse fascia and parietal peritoneum with no connection between cyst and other structures	Cystic mass at mesentery
Common location	In the lower third of the urachus	At the ileal mesentery
Malignancy	Sometimes	Sometimes
Recurrence	Frequent	Frequent
Treatment	Complete resection	Complete resection
Prognosis	Usually very good	Usually very good
